# Fatty Acid Composition from Olive Oils of Portuguese Centenarian Trees Is Highly Dependent on Olive Cultivar and Crop Year

**DOI:** 10.3390/foods10030496

**Published:** 2021-02-25

**Authors:** Nuno Rodrigues, Susana Casal, Teresa Pinho, Rebeca Cruz, António M. Peres, Paula Baptista, José Alberto Pereira

**Affiliations:** 1 Centro de Investigação de Montanha (CIMO), Instituto Politécnico de Bragança, Campus de Santa Apolónia, 5300-253 Bragança, Portugal; nunorodrigues@ipb.pt (N.R.); peres@ipb.pt (A.M.P.); pbaptista@ipb.pt (P.B.); 2 LAQV/REQUIMTE, Laboratory of Bromatology and Hydrology, Faculty of Pharmacy, University of Porto, Rua de Jorge Viterbo Ferreira, 228, 4050-313 Porto, Portugal; teresapinho847@gmail.com (T.P.); rcruz@ff.up.pt (R.C.)

**Keywords:** olive heritage, chemical characterization, chemometrics, crop year, monounsaturated fatty acids

## Abstract

The high proportions of monounsaturated fatty acids (MUFA) represent one of the most important technological and nutritional features of olive oils. The present study details the fatty acid (FA) composition of autochthonous cultivars (Lentisca, Madural, Redondal, Rebolã, Verdeal, and Verdeal Transmontana) produced from centenarian trees during five crop years (2013–2017). Olive cultivar highly influenced the FA composition, namely, oleic acid (70.3% for Madural to 80.7% for Redondal) and palmitic acid (10.4% for Lentisca to 13.5% for Verdeal). Similarly, crop year significantly influenced the individual FA contents. Principal component analysis of FA data enabled the unsupervised classification by cultivar and, within each cultivar, by crop year. Furthermore, the levels of nine individual FAs, together with the polyunsaturated fatty acid contents, selected using the simulated annealing algorithm, allowed for their correct classification, on the basis of linear discriminant analysis, according to the olive cultivar, with an overall sensitivity of 92%, for leave-one-out cross-validation procedure. Globally, the cultivar effect superimposed that of crop year, showing that some cultivars, such as Redondal and Verdeal Transmontana, have consistently high and homogeneous proportions of MUFA, indicating that they are worth exploring in terms of future selection of cultivars that are able to produce olive oils with increased nutritional value and that are less prone to oxidation.

## 1. Introduction

Extra virgin olive oil (EVOO) is a vegetable oil extracted from fresh and healthy olives (*Olea europaea* L.), by mechanical processes, respecting all good manufacturing practices of hygiene, safety, and temperature. EVOO is a natural product that can be consumed directly in its raw state and enjoys worldwide recognition, thanks to its nutritional value and beneficial effects on health [1]. The high demand of this product results from the high content of monounsaturated fatty acids (MUFA) and the presence of minor components such as phytosterols, squalene, vitamins, and antioxidants such as tocopherols and polar phenols [2], the latter supporting an authorized health claim by the European Food Safety Authority (EFSA) [3]. Extensive studies about the effect of olive oil on human health were developed in the frame of the European project PREDIMED [4].

Within the MUFA, oleic acid is the most abundant (55–83%), while palmitoleic and eicosenoic acid are present in reduced proportions. The remaining fatty acids include linoleic acid (3.5 to 21%), palmitic acid (7.5 to 20%), and linolenic acid (≤ 1%) [5]. Replacing saturated fats in the diet with unsaturated fats helps to maintain normal blood cholesterol levels [6]. Oleic acid seems to protect the human body against several types of diseases, having neurotrophic properties and slowing the progression of atheromatous lesion on the walls of the arteries by reducing low-density lipoproteins (LDL) oxidation [7,8].

However, the relative proportion of fatty acids in olive oil is not constant, but instead is dependent on several factors associated with cultivar genotype, as well as edaphoclimatic variables. Therefore, the nutritional and health benefits attributed to olive oil, as well as its sensorial properties, may vary greatly, depending on its fatty acid composition [9], as well as the proportions of other minor compounds, particularly antioxidants. The selection of cultivars that have consistently high oleic acid proportions, as well as a clearer understanding of the pedoclimatic factors that might influence oleic acid production in the olives, might help to provide interesting products from a nutritional and technological point of view. The effect of temperature on olive oil quality has been the subject of several studies, with it being known that the concentration of oleic acid is correlated with the average temperature [10,11], regulating fatty acid desaturases [12]. This fact was confirmed for cv. Arbequina, with a linear negative correlation between seasonal temperature (23–27 °C range) [13], as well as for cv. Arauco, where the percentage of oleic acid in the whole fruit (endocarp and mesocarp) decreased by 0.7% °C^−1^ with the increase of the average temperature in the 16–32 °C range during fruit growth [14]. Other environmental parameters, such as rainfall amounts and patterns, together with soil characteristics, agricultural practices, and fruit ripening have already been studied and are known to influence olive oil composition [10,15,16,17,18]. Still, most studies are only performed during a reduced number of years, reducing the accuracy of the conclusions regarding both cultivar and environmental effects on olive oil composition.

Some traditional olive cultivars, with reduced geographical expression but with a high biodiversity interest, may represent high sources of oleic acid that are worth knowing and preserving. In the north of Portugal, and more specifically in the region of Trás-os-Montes, there is a great wealth of cultivars, largely unknown and uncharacterized, and a large number of centenary olive trees, whose oils are potentially richer in bioactive compounds, possessing a well-balanced composition from nutritional and quality point of views.

In this context, during five consecutive crop years (2013–2017), the fatty acid composition of olive oils extracted from fruits produced by centenary trees belonging to six minor olive cultivars, some of them studied for the first time, were characterized. The effects of olive cultivar (cvs. Lentisca, Madural, Rebolã, Redondal, Verdeal, and Verdeal Transmontana) and crop year (2013–2017) were studied using different chemometric tools, aiming to identify the most promising ones in terms of oleic proportion and seasonal stability.

## 2. Materials and Methods

### 2.1. Sampling

For this work, an olive grove with centenarian trees, located in the northeast of Portugal, near Mirandela (Suçães, N 41°29′26.628′′; W 7°15′31.219′′), was selected. Around 20% of the trees were chosen, respecting the proportion of each olive cultivar in the grove. A total of 20 trees of different minor cultivars were identified and marked, namely, cvs. Lentisca (3 trees), Madural (3 trees), Rebolã (3 trees), Redondal (3 trees), Verdeal (2 trees), and Verdeal Transmontana (7 trees). From 2013 to 2017, in each crop year, and from each individual tree, approximately 3 kg of healthy olives was collected. In all crop years, harvest occurred at a similar maturity index (MI), between the MI 2 (fruit epidermis with red spots in less than half of the olive), and the MI 3 (fruit epidermis red or purple in more than half of the olive) [19]. More precisely, harvest occurred on 25 and 26 November in 2013, on 10 and 11 November in 2014, on 2 and 3 November in 2015, on 7 and 8 November in 2016, and on 13 and 14 November in 2017.

In the first 24 h after harvest, fruits were extracted in a pilot extraction plant with an Abencor analyzer (Comercial Abengoa S.A., Seville, Spain). Olives were milled, the obtained paste was homogenized, and about 900 g was transferred to the thermobeater unit (25 °C, 30 min) for malaxation at 25 °C. The mixture was centrifuged and decanted, and the olive oil was collected, filtered (Whatman paper n° 4 and anhydrous sulfate), and stored in the dark at room temperature in 125 mL dark bottles. The olive oils were analyzed for standard quality parameters [20], and all samples were classified as extra virgin olive oils—free acidity was below 0.32%, expressed in percentage of oleic acid; peroxides were below 5.2 mEqO2/kg olive oil; while for K232, the values were between 1.31 and 2.03, the K272 values varied from 0.11 to 0.23, and all samples were sensory classified as extra virgin olive oils without any sensory defects and with fruity median higher than 1. All the assays were carried out in triplicate within 2 months after extraction.

### 2.2. Fatty Acid Composition

Fatty acids were evaluated by gas chromatography (Chrompack CP 9001 with flame ionization detection, Varian, Middelburg, The Netherlands), after conversion to methyl esters using cold alkaline transesterification with methanolic potassium hydroxide solution following the recommended method for olive oil analysis [20]. The fatty acid profile was accomplished using a 50 m × 0.25 mm i.d. × 0.25 µm fused silica capillary column (SelectFAME, Agilent, Santa Clara, CA, USA) with helium as carrier gas at 110 kPa. The temperatures of the detector and injector were 250 and 230 °C, respectively, with an optimized temperature gradient for fatty acid complete separation. The fatty acid composition is expressed in relative percentage of each fatty acid on the total fatty acids eluting between myristic and lignoceric methyl esters as regulated [20]. A certified fatty acid methyl ester standard mixture (Supelco 37 Component FAME Mix) was used for identification and FID calibration purposes (Sigma, Madrid, Spain).

### 2.3. Statistical Analysis

One-way analysis of variance (one-way ANOVA) was applied to evaluate the existence of statistically significant effects of the tree cultivar or the crop year, on the fatty acid composition of olive oils extracted using the same production techniques, from olives collected at similar maturation stages, from centenarian olive trees grown in the same geographical area and under the same agricultural practices. Moreover, if a significant statistical effect was found (*p*-value < 0.050), the post hoc multi-comparison Tukey’s test was also further applied to identify the levels (i.e., olive tree cultivar or crop year) of each effect that was responsible for the detected significant effect. Boxplots were used to show the one-way ANOVA statistical results for each cultivar and all years evaluated, for saturated fatty acids, monounsaturated fatty acids, and polyunsaturated fatty acids.

The influences of olive tree cultivar (i.e., cvs. Lentisca, Madural, Rebolã, Redondal, Verdeal, or Verdeal Transmontana) or crop year (from 2013 to 2017) on the olive oil fatty acid composition of the olive oils extracted from olives of centenarian trees was also evaluated using principal component analysis (PCA), an unsupervised multivariate pattern recognition technique. For PCA, the fatty acid composition was centered and scaled minimizing data variability. The possibility of using the fatty acid composition of the oils to discriminate the different cultivars under study was further evaluated using linear discriminant analysis (LDA), a multivariate supervised pattern recognition technique, along with the simulated annealing (SA) variable selection algorithm. The use of the SA algorithm allowed for identifying which fatty acid parameters had the greatest discrimination power. For the LDA, the values of the different parameters were also centered and scaled, minimizing the variability of the data. The quality of discrimination performance was assessed by considering the correct classification rate for the pooled original data as well as for the internal single validation procedure (LOO-CV). The statistical analysis was performed using the Subselect [21,22,23] and MASS [24] packages of the open-source statistical program R (version 2.15.1), at a 5% significance level.

## 3. Results and Discussions

### 3.1. Effect of Olive Cultivar on Fatty Acid Composition

In the present work, the fatty acid composition of olive oils from minor cultivars, four of them not yet characterized (cvs. Lentisca, Rebolã, Redondal, and Verdeal) and two poorly known (cvs. Madural and Verdeal Transmontana), produced from centenarian trees, were characterized during five consecutive crop years (2013–2017) (Table 1). For the main fatty acids, all the samples of the different cultivars and throughout the five years of study fulfilled the levels established by the European Community Regulation for EVOO [20]. As expected, oleic acid (C_18:1_) was the main fatty acid found in all cultivars, and, combining the data from the five years, its average proportion ranged from 70.3% (cv. Madural) to 80.6% (cv. Redondal) (Table 1).

In all years, the proportions of oleic acid were statistically dependent on the olive cultivar (*p*-value < 0.0001). Three possible groups could be observed, one constituted by cvs. Verdeal Transmontana and Redondal olive oils, with the highest proportions, and mean values higher than 80%; one second group, with intermediate values, namely, cvs. Lentisca and Verdeal, had means of 78.0 and 75.8%, respectively; and a third group, with the lower proportions for the oils of cvs. Madural (70.3%) and Rebolã (72.9%) (Table 1). Palmitic acid (C_16:0_) was the second major fatty acid (Table 1). Consistently, the highest values of this fatty acid were obtained for cv. Verdeal oil, with a mean value of 13.5% combining the five years studied. Moreover, in general, the lowest levels were observed for cv. Lentisca (mean value of 10.4%). For this fatty acid, the proportions for cvs. Madural and Rebolã were similar, except for the 2017 crop year (Table 1). The proportions of the essential linoleic acid (C_18:2_), the third in order of abundance, with the exception for cv. Redondal, significantly differed between the studied cultivars. The highest proportions, and in general with statistical differences between cultivars, were observed for cv. Madural olive oils (mean value of 12.6%), followed by cv. Rebolã (mean value of 9.2%), totally opposite to the average proportion found for Redondal (2.19%) and Verdeal Transmontana (2.74%), with cv. Lentisca and cv. Madural in between (Table 1). Surprisingly, in cv. Rebolã olive oils, the amount of stearic acid (C_18:0_) (2.9%) was higher than the observed for linoleic acid (2.2%), contrarily to the observed for the other cultivars. Stearic acid ranged from 2 to 3% for all cultivars (Table 1). Consistently, the proportions for the essential linolenic acid (C_18:3_) was significantly higher for cv. Madural olive oils, with a mean value of 1.19%, whilst in the opposite place appeared cv. Lentisca (0.84%) and Verdeal Transmontana (0.76%) olive oils. The olive oils from cv. Madural surpassed the maximum value of ≤1.0 established by the European Community Regulation for EVOO [20], while cvs. Lentisca (0.84%), Rebolã (0.97%), Redondal (0.85%), Verdeal (0.91%), and Verdeal Transmontana (0.76%) were within this limit. Regarding palmitoleic acid (C_16:1_), the highest value was observed for cv. Verdeal olive oil (1.27%), which was the only cultivar with levels greater than 1% (Table 1). In general, the lowest proportions were obtained for cvs. Madural (mean 0.56%) and Verdeal Transmontana (0.64%). The highest proportions of arachidic acid (C_20:0_) were observed for Verdeal Transmontana (0.5%) and Redondal (0.48%) but were lower than the maximum (≤ 0.6%) established by the European Community Regulation [20]. Moreover, for eicosenoic acid (C_20:1_) none the cultivars reached the maximum proportion (≤ 0.4%) established by the same regulation.

The present results confirmed that the fatty acid composition is olive cultivar-dependent. Some variations were observed between them, being the established fatty acid profile for each cultivar in agreement with the literature data for diverse olive cultivars [16,18,25,26], although with different relative compositions. For example, for the main fatty acid (oleic acid), Xiang [26] found values ranging from 60.9 (cv. Barnea) to 74.0% (cv. Koreniki), and Wanga [27] from 62.2% (cv. Empeltre) to 74.5% (cv. Cornicabra), when studying fatty acid composition of different cultivars. However, the comparison is not straightforward since besides different cultivars being involved, also different geographic regions are evaluated. Indeed, Borges [16] verified that for cv. Arbequina olive oils, the geographic origin influences the fatty acid profile for the same cultivar. This variability factor was eliminated under the present study because all trees were grown under the same region. Therefore, the fatty acid variations observed are strictly dependent on the cultivar and the effect of climate on each cultivar.

When the fatty acids were grouped according to their degree of saturation, and considering the MUFA, five significantly different (*p* ≤ 0.05) groups could be identified in terms of relative abundance: cv. Redondal (82.1%) ≈ cv. Verdeal Transmontana (81.7%) > cv. Lentisca (79.1%) > cv. Verdeal (77.4%) > cv. Rebolã (74.3%) > cv. Madural (71.2%). An inverse trend was observed for polyunsaturated fatty acids (PUFA), with a relative abundance as follows: cv. Madural (13.8%) > cv. Rebolã (10.1%) > cv. Lentisca (7.0%) > cv. Verdeal (5.9%) > cv. Verdeal Transmontana (3.5%) ≈ cv. Redondal (3.0%). From a technological point of view, the presence of high PUFA proportions in vegetable oils decreases their resistance to oxidation, affecting both its shelf life and thermal performance. Significantly higher proportions for saturated fatty acids (SFA) were observed for cv. Verdeal (16.6%), followed by cv. Rebolã (15.5%), and the lowest proportions were observed for cv. Lentisca (13.8%). For SFA, cvs. Madural, Redondal, and Verdeal Transmontana presented similar relative proportions. The overall proportions of the different groups (saturated and unsaturated fatty acids) are of major relevance due to the related nutritional and technological impacts [28]. Indeed, besides the previously mentioned health effect of MUFA, the MUFA and PUFA proportions are also correlated with the oxidative stability of oils, with higher shelf life for olive oils richer in MUFA and usually lower for higher PUFA proportions. The proportions of MUFA are also known to be correlated with some sensory attributes, with established positive correlations with bitter attributes and negative ones with sweet attributes [29,30]. Moreover, Youssef [30] observed that a high content of SFA in olive oils lead to a higher viscosity and persistence on the mucous of the oral cavity, producing an effect known as “fatty sensation”, not sensory agreeable. All these reasons qualified fatty acid contents as quality indices of the oils during production, storage, and trade [29,30]. Globally, the results described in this work are in agreement with the literature data that considered the amounts of the different groups (MUFA, PUFA, and SFA) genetically dependent, and thus dependent of the cultivars (e.g., [25,26]).

### 3.2. Effect of Crop Year on the Fatty Acid Composition

For each cultivar and fatty acid, the relative proportions determined for each crop year are also given in Table 1. In general, with the exception of cv. Lentisca, the year of production showed a significant effect on the olive oil fatty acid composition for the different olive cultivars, but the cultivar effect was still prevalent. The proportion of oleic acid (C_18:1_), the major fatty acid, changed slightly with the crop year, and consistently, for the six cultivars, the values obtained in 2017 were significantly lower than the maximum values observed in other years. This fact could be related with the rain and temperatures observed in 2017 (Table 2), in agreement with literature findings [10,11,14]. In fact, among the five years covered in this study, 2017 was the most dried year, with a large period without rain, being considered a year of extreme drought. Considering the rainfall of August, September, and October, less than 30 mm of rain was observed in 2017, whereas, in the other years, the total rainfall of these months ranged from 100 to 180 mm (Table 2).

Moreover, the mean and maximum temperatures registered during October were higher in 2017. Temperature seems to be particularly determinant between flowering and harvest [16,18,31,32]. Oleic and palmitic acids are the fatty acids that seem to be more affected by the environmental conditions [33,34]. In the present study, palmitic acid was also significantly influenced by the crop year for all cultivars with the exception of cv. Lentisca (Table 1), although no marked trend was observed.

### 3.3. Cultivar and Crop Year Discrimination According to Fatty Acid Composition

**Figure 1:**
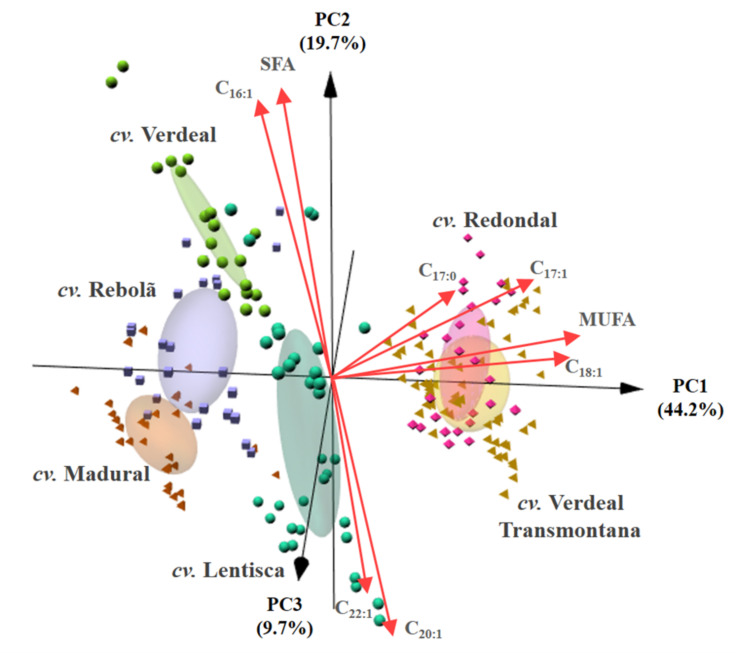
Principal component analysis (PC1: 44.2%, PC2: 19.7%, and PC3: 9.7%) based on the fatty acids profile (C_14:0_, C_16:0_, C_16:1_, C_17:0_, C_17:1_, C_18:0_, C_18:1_, C_18:2_, C_18:3_, C_20:0_, C_20:1_, C_22:0_, C_22:1_, C_24:0_, SFA, PUFA, and MUFA proportions): 3D biplot showing the unsupervised pattern recognition according to olive cultivar (cvs. Lentisca, Madural, Rebolã, Redondal, Verdeal, and Verdeal Transmontana) on the basis of fatty acids composition (%) found in olive oils obtained from olives collected in centenarian trees during five consecutive crop years (2013 to 2017).

As can be inferred from Figure 1, the overall olive oil fatty acid profiles allowed the unsupervised differentiation of the oils according to the olive cultivar. The visualization of the PCA-3D biplot, for which three first principal components (PCs) explained 73.6% of the data variability), clearly shows that olive oils from cvs. Lentisca, Madural, Rebolã, and Verdeal could be easily identified and distinguished from the other cultivars, being only a wide overplotting of olive oils from cvs. Redondal and Verdeal Transmontana. Among the 14 fatty acids identified and the total SFA, MUFA, and PUFA contents, the fatty acids that mostly contributed to the olive oils unsupervised classification by cultivar were C_16:0_ and total SFA, together with C_16:1_, C_17:1_, C_18:1_, C_20:1_, C_22:1_, C_16:1_, and total MUFA. As can be easily inferred, the composition in MUFA is the one that most contributed to the natural differentiation of the olive oils, pointing out the relevance of this fraction to the characterization of the studied olive oils. In more detail, the contents of C_16:0_ together with total MUFA and the individual C_17:1_ and C_18:1_ were the most relevant for the differentiation of oils of cvs. Redondal and Verdeal Transmontana from those of the other four cultivars (cvs. Lentisca, Madural, Rebolã, and Verdeal). On the other hand, the monounsaturated acids C_16:1_, C_20:1_, C_22:1_, and total SFA were those that mostly contributed to the differentiation of cvs. Lentisca, Madural, Rebolã, and Verdeal. To further verify the usefulness of the fatty acid profile to discriminate olive oils by cultivar and to identify the fatty acids that could be tentatively used as chemical biomarkers of each cultivar, we implemented a LDA coupled to with the SA algorithm (LDA-SA).

**Figure 2:**
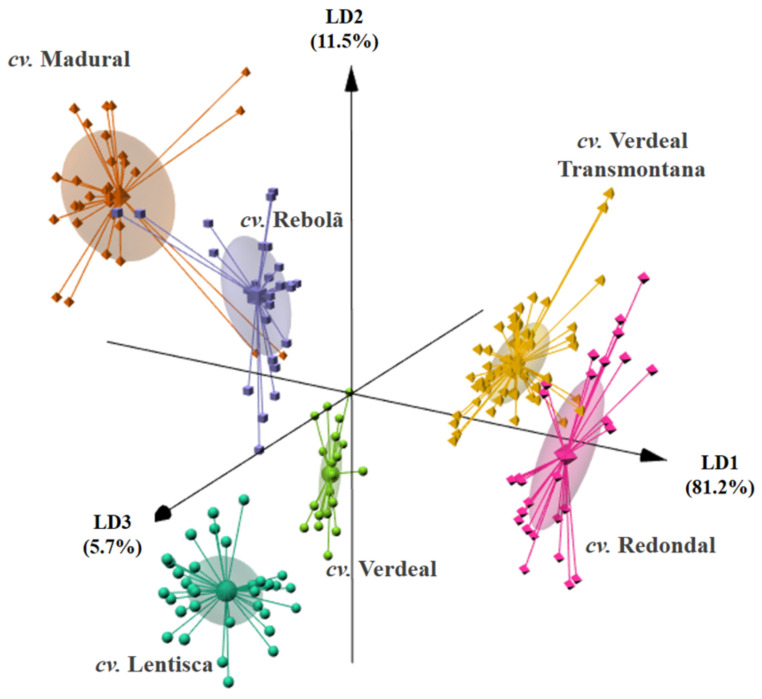
Linear discriminant analysis (LD1: 81.2%, LD2: 11.5%, and LD3: 5.7%): 3D biplot showing the discrimination of olive oil according to olive cultivar (cvs. Lentisca, Madural, Rebolã, Redondal, Verdeal, and Verdeal Transmontana) on the basis of the proportions (%) of nine fatty acids (C_16:0_, C_16:1_, C_17:0_, C_17:1_, C_18:0_, C_18:1_, C_20:0_, C_20:1_, and C_22:1_) and PUFA (selected using the simulated annealing algorithm) found in olive oils obtained from olives collected in centenarian trees during five consecutive crop years (2013 to 2017).

The results (Figure 2) showed that a LDA-SA model with three significant linear discriminant functions (LDs) allowed for 98.4% of the data variability to be explained (81.2%, 11.5%, and 5.7%), on the basis of the content of four saturated acids (C_16:0_, C_17:0_, C_18:0_, and C_20:0_), five monounsaturated compounds (C_16:1_, C_17:1_, C_18:1_, C_20:1_, and C_22:1_), and total PUFA. The established model allowed for the correct classification of 94% of the oils according to the cultivar for the original data grouped with a predicted correct classification rate of 92% for the LOO-CV procedure. These results confirmed that olive oil discrimination by cultivar can be satisfactorily accomplished using the oil fatty acid composition [16,18,25,30]. Nevertheless, it should be remarked that an overplot of some cultivars was observed, namely, between cvs. Madural and Rebolã, which could be tentatively attributed to the crop year effect since the studied oils were produced during five consecutive years (from 2013 to 2017).

**Figure 3:**
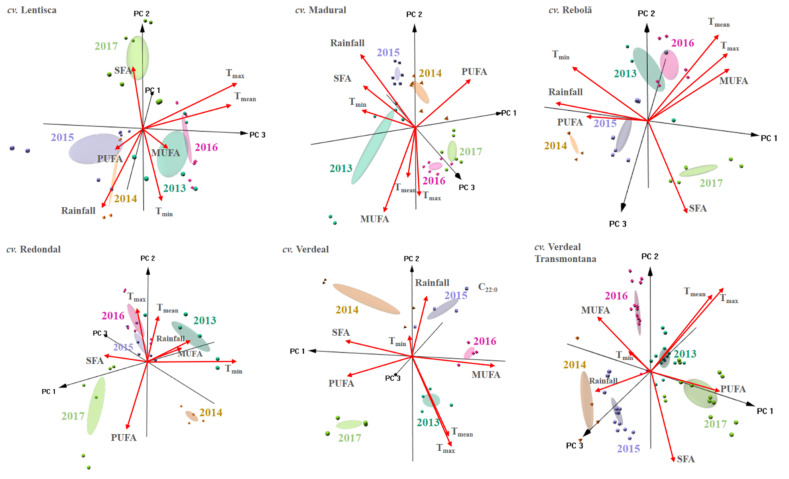
Principal component analysis for olive oils from each individual olive cultivar: 3D biplot showing the unsupervised pattern recognition according to crop year (2013 to 2017) on the basis of climatic data (minimum, mean, and maximum average temperatures (T_min_, T_mean_, and T_max_, respectively, in °C) and total rainfall (mm) recorded in the months of August, September, and October in Mirandela region, northern Portugal) and fatty acid profile (%) (C_14:0_, C_16:0_, C_16:1_, C_17:0_, C_17:1_, C_18:0_, C_18:1_, C_18:2_, C_18:3_, C_20:0_, C_20:1_, C_22:0_, C_22:1_, C_24:0_), MUFA, PUFA, and SFA proportions found in olive oils obtained from olives collected from centenarian trees of different cultivars (cvs. Lentisca, Madural, Rebolã, Redondal, Verdeal, and Verdeal Transmontana).

As mentioned, the main aim of this work was to study the fatty acid composition of centenarian trees belonging to different cultivars. As the study occurred during five consecutive years, for each cultivar evaluated, we also evaluated the possibility of using the fatty acid compositions together with the climatic data (Table 2) to identify the olive oils’ crop year. PCAs were carried out and, as can be seen from the PCA-3D biplots shown in Figure 3, for each cultivar, oils could be discriminated according to the crop year. In fact, as can be inferred from the overall plotted data, the five crop years studied could be naturally grouped into three subgroups classes. One subgroup included the oils produced in 2017, for which fatty acids composition was different from that of the oils produced in the other four studied years (2013–2016). The second subgroup comprised the oils produced in 2013 and 2016, with the last subgroup for the oils being produced in 2014 and 2015. These results clearly point out that, within each olive cultivar class, crop year has a huge impact on the fatty acid composition of produced olive oil. It should be remarked that the above-mentioned unsupervised subgroup formation could be tentatively related to the different climacteric conditions observed in 2014–2015 (high rainfall in September–October), 2013–2016 (low rainfall in August–September along with warmer months with higher minimum, mean, and maximum temperatures), and 2017 (dry and hot weather during all summer with the hottest temperature during this study), which can be inferred from the data shown in Table 2. Nevertheless, the relevance of the present works remains on the finding that fatty acid composition, although considering the inter-annual variations, showed a high steadiness level that allows for the differentiation of olive cultivars.

## 4. Conclusions

In the present work, oils obtained from centenarian specimens of six minority cultivars from Trás-os-Montes region, the second most important Portuguese olive region, were characterized regarding their fatty acid contents during five consecutive crop years, allowing for the strengthening of the findings achieved. Four of the six cultivars were studied for the first time (cvs. Lentisca, Rebolã, Redondal, and Verdeal). Regarding the fatty acid composition, we observed that oils from cvs. Redondal and Verdeal Transmontana were the richest in oleic acid, which could be indicative of a possible high shelf life and increased nutritional value. On the contrary, oils from cv. Madural had the lowest content in oleic acid, indicating a possible higher susceptibility to oxidation. The overall results also pointed out that both olive cultivar and crop year had marked effects on the oils fatty acid composition, which could be used as chemical biomarkers. Moreover, it was verified that the environmental conditions mainly influence oleic acid and linoleic acid contents and that some authentic cultivars are not within regulated limit for linolenic acid, highlighting for the pertinence of this short limit. The obtained results contribute to enhancing the scarce knowledge of olive heritage and may support the selection of olive cultivars for new plantations, on the basis of their potential to allow a more favorable fatty acid composition that concerns the nutritional and oil quality points of view.

## Figures and Tables

**Table 1:** Fatty acid composition (%) (mean ± standard deviation) of olive oils extracted from olives collected in centenarian trees of different cultivars (cvs. Lentisca, Madural, Rebolã, Redondal, Verdeal, and Verdeal Transmontana) during five consecutive crop years (2013 to 2017).

Figure	Year	cv. Lentisca	cv. Madural	cv. Rebolã	cv. Redondal	cv. Verdeal	cv. Verdeal Transmontana	*p*-Value *
Palmitic acid (C_16:0_)	2013	9.9 ± 0.4 ^a;C^	12.4 ± 0.6 ^a;A^	12.3 ± 0.7 ^a,b;A^	10.6 ± 0.4 ^a,b;B,C^	13.0 ± 0.1 ^a;A^	11.0 ± 0.3 ^a,b;B^	<0.0001
	2014	9.9 ± 0.4 ^a;C^	11.7 ± 0.3 ^b;B,C^	12.8 ± 0.5 ^a,b;B^	10.2 ± 0.3 ^b;C^	14.8 ± 2.1 a^;A^	11.1 ± 0.7 ^a,b;B,C^	<0.0001
	2015	10.6 ± 0.4 ^a;C^	12.6 ± 0.1 ^a;A^	12.9 ± 0.4 ^a;A^	10.9 ± 0.3 ^a;B,C^	12.8 ± 0.2 ^a;A^	11.3 ± 0.4 ^a;B^	<0.0001
	2016	10.2 ± 1.0 ^a;D^	11.3 ± 0.3 ^b;B,C^	12.0 ± 0.4 ^b;A,B;^	10.7 ± 0.4 ^a,b;C,D^	13.0 ± 0.1 ^a;A^	10.3 ± 0.5 ^c;D^	<0.0001
	2017	11.0 ± 1.4 ^a;B^	11.1 ± 0.1 ^b;B^	13.0 ± 0.2 ^a;A^	11.2 ± 0.2 ^a;B^	14.1 ± 0.2 ^a;A^	10.7 ± 0.6 ^b,c;B^	<0.0001
	*p*-value *	0.1650	<0.0001	0.0018	0.0022	0.0453	<0.0001	
	Mean (Min.–Max.)	10.4 (9.1–12.5)	11.8 (10.8–13.1)	12.6 (11.4–13.3)	10.8 (9.9–11.5)	13.5 (12.7–16.7)	10.8 (9.8–11.9)	
Palmitoleic acid (C_16:1_)	2013	0.68 ± 0.13 ^a;B^	0.76 ± 0.28 ^a;B^	0.71 ± 0.17 ^c;B^	0.80 ± 0.07 ^b;B^	1.23 ± 0.03 ^b;A^	0.71 ± 0.05 ^a;B^	<0.0001
	2014	0.61 ± 0.16 ^a;C^	0.48 ± 0.01 ^b;C^	1.30 ± 0.03 ^a;A^	1.03 ± 0.04 ^a;B^	1.39 ± 0.05 ^a;A^	0.58 ± 0.07 ^b,c;C^	<0.0001
	2015	0.79 ± 0.17 ^a;C^	0.57 ± 0.01 ª^,b;D^	0.99 ± 0.11 ^b;B^	0.70 ± 0.05 ^c;C,D^	1.25 ± 0.03 ^b;A^	0.68 ± 0.06 ^a,b;C,D^	<0.0001
	2016	0.69 ± 0.24 ^a;B,C^	0.48 ± 0.01 ^b,D^	0.83 ± 0.05 ^b,c;B^	0.76 ± 0.07 ^b,c;B,C^	1.18 ± 0.02 ^b;A^	0.59 ± 0.10 ^c;C,D^	<0.0001
	2017	0.79 ± 0.28 ^a;B,C^	0.53 ± 0.01 ^b;D^	0.83 ± 0.08 ^b,c;B^	0.79 ± 0.03 ^b,c;B,C^	1.41 ± 0.02 ^a;A^	0.61 ± 0.09 ^b,c;C,D^	<0.0001
	*p*-value *	0.5670	0.0033	<0.0001	<0.0001	<0.0001	0.0002	
	Mean (Min.–Max.)	0.72 (0.43–1.15)	0.56 (0.46–1.13)	0.92 (0.55–1.34)	0.80 (0.66–1.08)	1.29 (1.16–1.44)	0.64 (0.49–0.84)	
Stearic acid (C_18:0_)	2013	2.87 ± 0.40 ^a;B^	2.18 ± 0.23 ^c;C^	2.41 ± 0.25 ^a,b;B,C^	3.37 ± 0.33 ^a;A^	2.59 ± 0.02 ^a;B,C^	2.65 ± 0.11 ^b;B^	<0.0001
	2014	2.69 ± 0.23 ^a;A^	2.44 ± 0.04 ^b;A,B,C^	1.84 ± 0.04 ^c,D^	2.27 ± 0.15 ^c,C^	2.35 ± 0.09 ^b;B,C^	2.60 ± 0.14 ^b;A,B^	<0.0001
	2015	2.54 ± 0.29 ^a;A,C^	2.25 ± 0.04 ^b,c;C,D^	2.07 ± 0.09 ^b,c;D^	2.84 ± 0.27 ^b;A^	2.45 ± 0.03 ^b;B,C^	2.68 ± 0.14 ^b;A,B^	<0.0001
	2016	2.70 ± 0.18 ^a;A,B^	2.31 ± 0.10 ^b,c,C^	1.97 ± 0.10 ^b,c,C^	2.88 ± 0.42 ª^,b;A^	2.38 ± 0.03 ^b,B,C^	2.70 ± 0.25 ^b;A,B^	<0.0001
	2017	2.92 ± 0.35 ^a;A,B,C^	2.81 ± 0.23 ª^;B,C^	2.74 ± 0.45 ^a;B,C^	3.16 ± 0.09 ª^,b;A,B^	2.45 ± 0.02 ^b,C^	3.35 ± 0.45 ^a;A^	0.0004
	*p*-value *	0.1360	<0.0001	<0.0001	<0.0001	<0.0001	<0.0001	
	Mean (Min.-Max.)	2.75 (2.11–3.42)	2.40 (1.89–3.10)	2.22 (1.80–3.37)	2.92 (2.13–3.77)	2.45 (2.26–2.63)	2.83 (2.33–3.89)	
Oleic acid (C_18:1_)	2013	77.0 ± 2.5 ^a;B^	70.5 ± 1.8 ^a,b;C^	72.6 ± 2.5 ^a,b;C^	80.4 ± 0.3 ^b;A^	76.8 ± 0.2 ^a;B^	80.4 ± 0.2 ^b;A^	<0.0001
	2014	79.2 ± 1.1 ^a;B^	70.9 ± 0.4 ^a;D^	70.0 ± 0.5 ^b;D^	81.6 ± 0.1 ^a;A^	74.2 ± 2.1 ^b;C^	80.8 ± 0.9 ^a,b;A,B^	<0.0001
	2015	77.6 ± 2.4 ^a;B^	69.0 ± 0.5 ^b;D^	73.3 ± 1.8 ^a,b;C^	80.8 ± 0.3 ^a,b;A^	76.8 ± 0.3 ^a;B^	80.2 ± 0.5 ^b;A^	<0.0001
	2016	79.2 ± 1.2 ^a;B^	71.2 ± 0.3 ^a,E^	75.3 ± 0.6 ^a,D^	80.9 ± 0.7 ^a,b;A^	76.9 ± 0.1 ^a,C^	81.3 ± 0.4 ^a;A^	<0.0001
	2017	77.1 ± 1.2 ^a;B^	69.7 ± 0.6 ^a,b;D^	72.4 ± 2.0 ^b;C^	79.3 ± 1.0 ^c;A^	74.1 ± 0.4 ^b;C^	79.3 ± 0.6 ^c;A^	<0.0001
	*p*-value *	0.0856	0.0022	0.0019	<0.0001	0.0005	<0.0001	
	Mean (Min.–Max.)	78.0 (74.2–80.6)	70.3 (68.3–72.9)	72.9 (69.6–75.8)	80.6 (78.1–81.8)	75.8 (72.4–77.1)	80.4 (78.3–82.1)	
Linoleic acid (C_18:2_)	2013	7.27 ± 2.77 ^a;B,C^	11.77 ± 2.18 ^b;A^	9.68 ± 2.81 ^a,b:A,B^	2.10 ± 0.11 ^b;C^	4.32 ± 0.05 ^c;C^	2.76 ± 0.15 ^b;C^	<0.0001
	2014	5.56 ± 1.63 ^a;B^	12.08 ± 0.31 ª^,b;A^	11.74 ± 0.92 ^a;A^	2.12 ± 0.01 ^b;C^	5.25 ± 0.04 ^b;B^	2.31 ± 0.12 ^c;C^	<0.0001
	2015	6.40 ± 2.70 ^a;C^	13.17 ± 0.42 ^a,b;A^	8.65 ± 1.32 ª^,b;B^	1.90 ± 0.12 ^b;D^	4.76 ± 0.24 ^b,c;C^	2.55 ± 0.19 ^b,c;D^	<0.0001
	2016	5.33 ± 2.03 ^a;C^	12.39 ± 0.49 ª^,b;A^	7.82 ± 0.45 ^b;B^	2.06 ± 0.16 ^b;D^	4.56 ± 0.12 ^c,C^	2.55 ± 0.06 ^b,c;D^	<0.0001
	2017	6.13 ± 2.46 ^a;C^	13.63 ± 0.28 ª^;A^	8.98 ± 2.16 ª^,b;B^	2.75 ± 0.48 ^a;D^	6.08 ± 0.48 ^a;C^	3.22 ± 0.34 ^a;D^	<0.0001
	*p*-value *	0.6370	0.0253	0.0227	<0.0001	<0.0001	<0.0001	
	Mean (Min.–Max.)	6.15 (3.33–9.73)	12.61 (8.90–14.0)	9.17 (6.06–12.55)	2.19 (1.77–3.34)	4.99 (4.27–6.50)	2.74 (2.16–3.76)	
Linolenic acid (C_18:3_)	2013	0.93 ± 0.20 ^a;B,C^	1.27 ± 0.19 ^a;A^	1.17 ± 0.23 ^a;A,B^	0.90 ± 0.07 ^a;C^	0.96 ± 0.06 ª^;B,C^	0.81 ± 0.06 ^a;C^	<0.0001
	2014	0.82 ± 0.13 ^a;C^	1.20 ± 0.16 ^a;A^	1.07 ± 0.02 ^a,b;A,B^	0.90 ± 0.01 ^a;B,C^	0.89 ± 0.05 ª^,b;B,C^	0.74 ± 0.04 ^a,b;C^	<0.0001
	2015	0.89 ± 0.23 ^a;B^	1.23 ± 0.06 ^a;A^	0.92 ± 0.04 ^b,c;B^	0.88 ± 0.05 ^a,b;B,C^	0.87 ± 0.01 ^b;B,C^	0.76 ± 0.04 ^a,b,C^	<0.0001
	2016	0.74 ± 0.09 ^a;C^	1.15 ± 0.02 ^a;A^	0.92 ± 0.12 ^b,c;B^	0.80 ± 0.02 ^b,c;B,C^	0.88 ± 0.02 ^b;B^	0.74 ± 0.05 ^b;C^	<0.0001
	2017	0.83 ± 0.14 ^a;B,C^	1.11 ± 0.03 ^a;A^	0.86 ± 0.04 ^c;B,C^	0.77 ± 0.04 ^c;C,D^	0.94 ± 0.03 ^a,b;B^	0.74 ± 0.07 ^b;D^	<0.0001
	*p*-value *	0.2930	0.1610	0.0014	<0.0001	0.0134	0.0051	
	Mean (Min.–Max.)	0.84 (0.65–1.19)	1.19 (1.00–1.41)	0.97 (0.80–1.37)	0.85 (0.73–0.97)	0.91 (0.84–1.01)	0.76 (0.65–0.89)	
Arachidic acid (C_20:0_)	2013	0.41 ± 0.04 ^a;B^	0.35 ± 0.03 ^b;C^	0.37 ± 0.03 ^b;B,C^	0.51 ± 0.03 ^a,A^	0.38 ± 0.02 ^a;B,C^	0.48 ± 0.02 ^b,A^	<0.0001
	2014	0.37 ± 0.06 ^a;A^	0.48 ± 0.15 ^a;A^	0.35 ± 0.01 ^b;A^	0.41 ± 0.03 ^b;A^	0.33 ± 0.03 ^b;A^	0.49 ± 0.03 ^a,b;A^	0.0279
	2015	0.35 ± 0.05 ^a;B^	0.34 ± 0.01 ^b;B^	0.38 ± 0.02 ^b;B^	0.48 ± 0.04 ^a;A^	0.37 ± 0.02 ^a,b;B^	0.49 ± 0.02 ^b;A^	<0.0001
	2016	0.39 ± 0.06 ^a;B^	0.36 ± 0.01 ^b;B^	0.36 ± 0.01 ^b;B^	0.48 ± 0.05 ^a;A^	0.39 ± 0.01 ^a;B^	0.50 ± 0.03 ^a,b;A^	<0.0001
	2017	0.40 ± 0.07 ^a;C^	0.37 ± 0.02 ^a,b,C^	0.41 ± 0.03 ^a;B,C^	0.49 ± 0.02 ^a;A,B^	0.36 ± 0.02 ^a,b;C^	0.53 ± 0.05 ^a;A^	<0.0001
	*p*-value *	0.3670	0.0088	0.0005	0.0044	0.0092	0.0030	
	Mean (Min.–Max.)	0.39 (0.29–0.48)	0.38 (0.30–0.68)	0.38 (0.34–0.44)	0.48 (0.39–0.54)	0.37 (0.30–0.41)	0.50 (0.44–0.61)	
Eicosenoic acid (C_20:1_)	2013	0.36 ± 0.10 ^a;A^	0.34 ± 0.03 ^a;A^	0.30 ± 0.03 ª^,b;A,B^	0.33 ± 0.03 ^b,c;A^	0.24 ± 0.01 ^b,c;B^	0.32 ± 0.02 ^b,c;A^	0.0063
	2014	0.34 ± 0.09 ^a;A^	0.34 ± 0.01 ^a;A^	0.32 ± 0.01 ª^;A,B^	0.39 ± 0.01 ^a;A^	0.25 ± 0.02 ^a;B^	0.35 ± 0.02 ^a;A^	0.0014
	2015	0.33 ± 0.10 ^a;A,B^	0.32 ± 0.01 ^a,b;A,B^	0.30 ± 0.01 ª^;A,B^	0.36 ± 0.02 ^a,b;A^	0.26 ± 0.01 ^a,b;B^	0.34 ± 0.01 ^a,b;A^	0.0230
	2016	0.30 ± 0.08 ^a;A,B^	0.32 ± 0.00 ^a,b;A^	0.31 ± 0.04 ª^;A,B^	0.33 ± 0.01 ^b,c;A^	0.24 ± 0.01 ^a,b;B^	0.32 ± 0.01 ^b,c;A^	0.0215
	2017	0.30 ± 0.08 ^a;A^	0.30 ± 0.01 ^b;A^	0.26 ± 0.01 ^b;A,B^	0.32 ± 0.00 ^c;A^	0.22 ± 0.01 ^c;B^	0.31 ± 0.02 ^c;A^	0.0004
	*p*-value *	0.7560	0.0038	0.0040	<0.0001	0.0006	<0.0001	
	Mean (Min.-Max.)	0.32 (0.22–0.45)	0.32 (0.28–0.39)	0.30 (0.24–0.37)	0.34 (0.29–0.40)	0.24 (0.21–0.28)	0.33 (0.28–0.36)	

In each column and for each olive cultivar, different lower-case letters mean significant statistical differences of the fatty acid relative percentage with the crop year, at a 5% significance level (*p*-value < 0.05), according to the multiple comparison Tukey’s HSD (honestly significant difference) test. In each line and for each crop year, different caps letters mean significant statistical differences of the fatty acid relative percentage with the olive cultivar, at a 5% significance level (*p*-value < 0.05), according to the multiple comparison Tukey’s HSD test. * *p*-value < 0.05 meaning that the mean value of the evaluated fatty acid relative percentage of at least one olive cultivar (for the lines) or of at least one crop year (for the columns) differs from the others, according to the one-way ANOVA results (in this case multiple-comparison tests were performed).

**Table 2:** The mean temperature (°C) (minimum, mean, and maximum) and total rainfall (mm) in the months of August, September, and October of Mirandela region (northern Portugal) over five years (2013–2017).

Year–Month	Temperature Records (°C)			Rainfall (mm)
	Minimum	Mean	Maximum	
2013				
August	16.8 ± 2.2	25.6 ± 9.3	34.4 ± 3.4	0.0
September	14.2 ± 2.2	22.4 ± 8.8	30.5 ± 3.8	30.0
October	11.0 ± 3.8	16.4 ± 6.4	21.9 ± 2.9	111.4
2014				
August	14.3 ± 2.6	22.7 ± 8.9	31.2 ± 2.6	0.4
September	14.5 ± 4.5	21.0 ± 7.6	27.6 ± 4.5	84.8
October	10.9 ± 3.0	17.1 ± 6.8	23.2 ± 3.0	77.2
2015				
August	14.5 ± 2.6	23.2 ± 9.4	31.9 ± 4.4	20.2
September	11.9 ± 3.0	19.5 ± 8.3	27.1 ± 3.5	50.4
October	9.8 ± 3.3	15.2 ± 6.2	20.6 ± 2.7	109.4
2016				
August	15.8 ± 2.4	25.1 ± 9.9	34.5 ± 3.2	32.2
September	12.9 ± 2.8	21.4 ± 9.6	30.0 ± 5.5	24.2
October	9.9 ± 2.7	16.3 ± 7.2	22.6 ± 3.8	41.6
2017				
August	15.0 ± 2.8	24.4 ± 10.0	33.8 ± 3.4	1.2
September	10.8 ± 3.1	19.9 ± 9.6	28.9 ± 3.1	0.2
October	8.9 ± 3.7	17.7 ± 10.0	26.5 ± 5.3	28.6

## Data Availability

Data are contained within this article.
